# Facility‐based HIV self‐testing strategies may substantially and cost‐effectively increase the number of men and youth tested for HIV in Malawi: results from an individual‐based mathematical model

**DOI:** 10.1002/jia2.26020

**Published:** 2022-10-17

**Authors:** Brooke E. Nichols, Alexandra de Nooy, Mariet Benade, Kelvin Balakasi, Misheck Mphande, Gabriella Rao, Cassidy W. Claassen, Shaukat Khan, Christian Stillson, Colin A. Russell, Naoko Doi, Kathryn Dovel

**Affiliations:** ^1^ Department of Global Health, School of Public Health Boston University Boston Massachusetts USA; ^2^ Health Economics and Epidemiology Research Office, Department of Internal Medicine, Faculty of Health Sciences, School of Clinical Medicine, University of the Witwatersrand Johannesburg South Africa; ^3^ Department of Medical Microbiology Amsterdam University Medical Centre Amsterdam the Netherlands; ^4^ Foundation for Innovative New Diagnostics Geneva Switzerland; ^5^ Right to Care Johannesburg South Africa; ^6^ Partners in Hope Lilongwe Malawi; ^7^ Tufts University Boston Massachusetts USA; ^8^ Center for International Health, Education, and Biosecurity University of Maryland School of Medicine Baltimore Maryland USA; ^9^ Division of Infectious Diseases, Department of Medicine University of Zambia School of Medicine Lusaka Zambia; ^10^ Clinton Health Access Initiative Boston Maryland USA; ^11^ Division of Infectious Diseases, Department of Medicine University of California Los Angeles David Geffen School of Medicine Los Angeles California USA

**Keywords:** cost‐effectiveness analysis, healthcare facilities, HIV self‐testing, mathematical modelling, priority populations, sub‐Saharan Africa

## Abstract

**Introduction:**

Malawi is rapidly closing the gap in achieving the UNAIDS 95‐95‐95 targets, with 90% of people living with HIV in Malawi aware of their status. As we approach epidemic control, interventions to improve coverage will become more costly. There is, therefore, an urgent need to identify innovative and low‐cost strategies to maintain and increase testing coverage without diverting resources from other HIV services. The objective of this study is to model different combinations of facility‐based HIV testing modalities and determine the most cost‐effective strategy to increase the proportion of men and youth testing for HIV.

**Methods:**

A data‐driven individual‐based model was parameterized with data from a community‐representative survey (socio‐demographic, health service utilization and HIV testing history) of men and youth in Malawi (data collected August 2019). In total, 79 different strategies for the implementation of HIV self‐testing (HIVST) and provider‐initiated‐testing‐and‐counselling at the outpatient department (OPD) were evaluated. Outcomes included percent of men/youth tested for HIV in a 12‐month period, cost‐effectiveness and human resource requirements. The testing yield was assumed to be constant across the scenarios.

**Results:**

Facility‐based HIVST offered year‐round resulted in the greatest increase in the proportion of men and youth tested in the OPD (from 45% to 72%–83%), was considered cost‐saving for HIVST kit priced at $1.00, and generally reduced required personnel as compared to the status quo. At higher HIVST kit prices, and more relaxed eligibility criteria, all scenarios that considered year‐round HIVST in the OPD remained on the cost‐effectiveness frontier.

**Conclusions:**

Facility‐based HIVST is a cost‐effective strategy to increase the proportion of men/youth tested for HIV in Malawi and decreases the human resource requirements for HIV testing in the OPD—providing additional healthcare worker time for other priority healthcare activities.

## 1 INTRODUCTION

Among Malawian people living with HIV, 90% knew their status, 87% of those who knew their status were on treatment and 92% of those on treatment had achieved viral suppression—rapidly closing the gap in achieving the UNAIDS 95‐95‐95 by 2025 targets [1]. However, these successes are inconsistent across sub‐populations with groups like men and youth showing disparities in the treatment cascade [2, 3]. Innovative strategies are needed to address these disparities; however, effective interventions become more costly as we approach epidemic control [4]. There is an urgent need to identify strategies to maintain and increase testing coverage to close the final testing gap for men and youth without diverting resources from other HIV services.

Routine HIV testing often misses men and youth in Malawi [5]. Men are often missed as their frequent entry point into health facilities—outpatient departments (OPDs)—rarely results in routine provider‐initiated‐testing‐and‐counselling (PITC), unlike family planning or antenatal entry points frequented by women [6, 7]. Thus, for men, accessing testing often requires more effort and greater indirect costs (e.g. through missed income) [8]. One novel strategy to improve testing coverage in OPD is HIV self‐testing (HIVST), whereby individuals conduct and interpret their own HIV test [9]. Clients who disclose a reactive HIVST result are then referred for professional‐use testing following the national testing algorithm. HIVST is a promising strategy for scale‐up in Malawi and across similar settings due to its simple procedure, low staffing requirements, high uptake and acceptability among men and youth, and low risk for adverse events [10]. Among outpatients in Malawi, facility‐based HIVST has proven to increase testing coverage compared to PITC [11]. Facility‐based HIVST may also be less costly and more effective than community‐based self‐testing strategies, as both men and youth do attend health facilities, and more specifically, OPD [6, 12]. Thus, a combination of HIVST and PITC within OPD could vastly increase testing coverage in Malawi and other similar settings, without incurring substantial additional cost.

Only a handful of studies have evaluated the impact of facility‐based HIVST, and most are implemented over a brief time period [13, 14]. It is possible that there is a reduction in the effectiveness of a facility‐based HIVST strategy as the population to be tested saturates. Additionally, these trials did not assess the proportion of community members likely to benefit from a facility‐based HIVST strategy, leaving a critical gap in understanding the potential reach of facility HIVST at the population level. Fortunately, our recent community‐representative survey of men and youth in Malawi has demonstrated that, while the proportion of men and youth who tested for HIV in the past year is relatively low (45%), the majority of those surveyed had visited a health facility in the past year (82%) as either a client (61% of visits) or a guardian (to support the healthcare of others) (39% of visits) [6]. Most attended OPD (84% of visits) [6]. This indicates that a facility‐based testing strategy, with sufficient coverage of attendees, would improve community‐level testing among men and youth since most individuals regularly attend facilities.

Through the creation of a data‐driven individual‐based model, parameterized with survey data of a community‐representative survey of men and youth in Malawi, we modelled the impact of using facility‐based HIV testing modalities to determine the most cost‐effective strategy to increase the proportion of men and youth testing for HIV.

## 2 METHODS

### 2.1 Study population

This individual‐based simulation model was parameterized using the results of a community‐representative survey of 1180 Malawian men (ages 15–64 years) and 300 young women (ages 15–24 years). The survey was conducted from August to October 2019 across 36 randomly selected villages in Central and Southern Malawi using a multi‐staged sampling design. The parent study was designed to assess utilization of facility‐based health services over the past 2 years, offer and uptake of HIV testing services, reasons for testing or not and willingness to use an HIV self‐test. Additional details regarding the parent study have been published elsewhere [6].

Eligibility criteria included: (1) aged 15–64 years for men or aged 15–24 for women; (2) current resident of the participating village; and (3) spent >15 nights within the village in the past 30 days. Those who self‐reported as ever testing HIV positive were excluded since they fall outside the target population for HIV testing strategies. Random selection was stratified by village (*n*∼45 per village, although some villages had fewer than 45 men due to small village size) and age categories: young men (15–24 years, *n* = 300); middle‐aged men (25–39 years, *n* = 425); older men (40+ years, *n* = 425); and young women (15–24 years, *n* = 300).

### 2.2 Model development

Characteristics were assigned to each individual in the model using survey results (*n* = 1480). Characteristic variables included age, sex and numerous questions regarding facility attendance history, such as the date and reason for the visit, whether HIV testing was offered and/or accepted, and whether the visit was as a client or a guardian. Therefore, each simulation reflects the demographics, patient journey and HIV testing history of individuals from this community representative survey. This allows us to understand the likely result of different facility‐based HIV testing strategies at different times. The simulations were scaled by a factor of 100 to aid interpretability for a total simulated population of 148,000.

### 2.3 Facility attendance history

Survey data included facility attendance dates for the four most recent visits within the past 4 years (whether as client or guardian), as well as the total number of visits over a 24‐month period prior to the survey. Given likely recall bias for visits >12 months ago, this analysis only included visits within the last 12 months. For every visit date assigned, the visit reason, whether the visit was as a client or a guardian, was recorded and assigned to the individual in the model. The individual simulations were programmed in MATLAB v9.7 (Natick, MA).

### 2.4 Human resource calculations

We estimated the number of healthcare worker hours required to implement each facility‐based testing scenario (time required per test reported in Table 1). Calculations were based on the staff time required to complete all tests within a given month, assuming an equal distribution of tests across all weekdays. Using different algorithms for PITC and HIVST, the total person time, and subsequent daily staff requirement, was determined for each scenario assuming 6 hours of patient–provider interaction per day per staff member and 21.5 working days per month. The number of healthcare workers required in the month of peak testing was then calculated to ensure that the described staff requirements would be sufficient for all months under consideration. The PITC and HIVST staff time algorithms are described below.

**Table 1:** Key HIV testing cost and resource assumptions [14, 15]

Resource	Assumption
**Key resource assumptions**	
**Personnel (health diagnostic assistant) time**	
HIV‐negative PITC	20 minutes
HIV‐positive PITC	50 minutes
HIV‐negative HIVST	3–6 minutes^a^
HIV‐positive HIVST	53–56 minutes^b^
**Key cost assumptions**		
**Personnel cost**	*Unit*	*Unit cost*
Health diagnostic assistant salary	Monthy	$117.30
**HIV test kit costs**	*Unit*	*Unit cost*
Determine (first PITC test)	Test	$0.80
Unigold (confirmation PITC test)	Test	$1.00
**HIV self‐test kit**		
*Low*	Test	$1.00
*Medium*	Test	$1.40
*High*	Test	$1.80
***All‐inclusive cost per test*** ^**c**^		
**Provider testing (all‐inclusive cost)**	*Unit*	*Unit cost*
PITC positive	Test	$2.70
PITC negative	Test	$1.46
**HIV self‐testing (all‐inclusive cost)**		**Base case**	**Medium**	**Low**
HIVST positive	Test	$4.56	$4.16	$3.76
HIVST negative	Test	$1.86	$1.46	$1.06

^a^ Depending on the size of group for HIV self‐test demonstration.

^b^ Time for testing negative HIV self‐test *plus* time for testing positive in the provider‐initiated‐testing‐and‐counselling algorithm.

^c^ Includes additional consumables, equipment and overhead [14, 15].

### 2.5 PITC staff time

It was assumed that each negative and positive HIV test requires 20 and 50 minutes of staff time, respectively [15]. Overall, PITC staff time was taken as the summation of person‐time for the number of positive and negative PITC tests assigned to the cohort across the 12 months. From the community representative survey, 15% of individuals refused PITC HIV testing when offered. This was assigned during simulations where it was assumed that individuals of this group would not be successfully tested by PITC [6].

### 2.6 HIVST staff time

Self‐testing scenarios assumed an implementation through group information sessions, each hosted by one staff member, followed by self‐test kit distribution. Sessions were assumed to consist of a 30‐minute HIVST demonstration, 10 minutes for questions and an additional 2 minutes of test kit distribution per individual in the group [14]. We ranged the number of HIVST demonstrations and question sessions per day between 2 and 6, with their person‐time divided by the daily average number of people tested that respective month. The test kit distribution time was multiplied by the average number of people testing across the month. Those who tested positive with HIVST were then assumed to enter the PITC testing algorithm and, therefore, an additional 50 minutes of testing person‐time was required. For HIVST scenarios, 86% of adult men, 88% of young men and 88% of young women were assumed to accept an HIVST based on the community survey. We assumed that HIVST refusers were offered a test through PITC, with 96% of these individuals accepting PITC [6].

### 2.7 Testing scenarios

We considered a set of scenarios to review the impact of different facility‐based testing interventions. These scenarios were based on implementation practices of both PITC and facility‐based HIVST pathways. A key limitation of relying on PITC for facility‐based testing is severalfold: relying on providers to implement PITC per guidelines (offer HIV testing at every visit), and the personnel required to implement these guidelines. Facility‐based HIVST, as implemented to date [16], has taken advantage of the long queues that are typically present to access health services in Malawi. While queuing for healthcare, individuals can opt to self‐test in private areas of the healthcare clinic, and subsequently discuss their test results with their providers. This method of facility‐based HIVST has shown high levels of linkage to care compared to other HIVST methods [12, 14].

Therefore, the scenarios included variations of the following:

1. Varying coverage of standard (professional use) PITC and HIVST from 5% to 90%
2. Targeted to OPD clients, guardians or both
3. Targeted to different periods (3 months with highest OPD volume, 9 months with low OPD volume or full 12 months)

A set of 79 mutually exclusive intervention combinations of the above three factors were considered (Table 2). Assignment of tests at each coverage level was performed using random number generation. For example, a baseline client coverage of 50% PITC would be the percentage of all visits, at random, that clients were offered PITC. Scenarios consider how adapting testing from baseline may impact the number of positive HIV individuals identified and the associated costs. The final number of people tested is the number offered tests multiplied by the uptake of testing at the individual level, parameterized from the community survey. Scenarios where just 3 months were targeted for intervention were evaluated to test whether a more parsimonious testing algorithm could result in a similar number of people being diagnosed. In Malawi, there is typically a 3‐month period each year with an increase in OPD visits due to influenza infections or malaria, and these HIV testing scenarios reflect implementation during this time. For the full facility‐based HIVST scenarios, where HIVST is offered year‐round, we also assessed different criteria for eligibility to test (defined as no criteria and 12+ months, 6+ months and 3+ months since the last test). These screening criteria limit or allow repeat testing for those who previously self‐reported a negative test result.

**Table 2:** Summary of combinations of facility‐based HIV self‐testing and provider‐initiated‐testing‐and‐counselling scenarios tested

			Provider‐initiated‐testing‐and‐counselling (PITC)						HIV self‐testing (HIVST)			
			12 months		9 months^a^		3 months^b^		12 months		3 months^b^	
Testing type Number of months implemented			Person tested	Percentage^c^	Person tested	Percentage^c^	Person tested	Percentage^c^	Person tested	Percentage^c^	Person tested	Percentage^c^
Scenario	1	Client PITC	Client	5–90%								
	2	Client‐guardian PITC	Both^d^	5–90%								
	3	Time‐differentiated client‐guardian PITC			Both	10%	Both	15–90%				
	4	Client‐guardian PITC with high‐volume HIVST			Both	5–90%	Both	HIVST refusers^e^			Both	100%
	5	Client PITC with high‐volume guardian HIVST	Client	5–90%							Guardian	100%
	6	Differentiated PITC and high‐volume guardian HIVST			Both	5–90%	Client	5–90%			Guardian	100%
	7	Differentiated PITC and high‐volume client HIVST			Both	5–90%	Guardian	5–90%			Client	100%
	8	Self‐testing only	HIVST refusers^e^					Both	100%			

^a^ Low‐volume outpatient department months.

^b^ High‐volume outpatient department months.

^c^ Percentage of all visits covered with a test.

^d^ Both indicate that clients and guardians were eligible for testing in the given scenario.

^e^ Provider‐initiated‐testing‐and‐counselling only offered to those who refused HIV self‐testing.

Within the model design and for each scenario, there are noted elements which used randomization to assign tests to visits. As such, to achieve a better overview of what a typical implementation may look like, each scenario was iterated 100 times and the average results for all iterations were exported for analysis.

### 2.8 Scenario costing and cost‐effectiveness analysis

To calculate the total costs for each scenario, we accounted for the full cost of test kits, including professional‐use tests prescribed by Malawi's HIV testing algorithm, Determine HIV 1/2 (Alere), Uni‐Gold HIV (Trinity Biotech) and OraQuick HIVST (OraSure), staff time, consumables and overhead estimated from previous work [14, 15]. The unit costs for positive and negative tests by modality are presented in Table 1. To determine cost‐effectiveness, we calculated the incremental cost‐effectiveness ratio of each scenario at different HIVST kit prices—given that the kit price can affect the order of scenarios on the cost‐effectiveness frontier. To enable comparability to the literature, we define effectiveness as individuals newly diagnosed with HIV [15, 16]. We have set a constant 2.5% testing yield, as seen in the community survey and facility‐based HIVST trial, across scenarios to enable the use of this outcome measure [14]. We considered standard PITC costs and three different price points for HIVST kit to reflect the shifting market: $1.80 (base case), $1.40 and $1.00 per test kit. Testing scenarios that are on the cost‐effectiveness frontier are reported separately. Costs are reported in 2018 USD collected as part of a previous facility‐based HIVST trial in Malawi [14, 15].

### 2.9 Ethics

The National Health Sciences Review Committee of Malawi (number 2338) and the University of California Los Angeles Institutional Review Board (number 20–001606) approved the study activities. All eligible individuals completed a written informed consent form immediately following screening procedures. For individuals between 15 and 18 years of age, guardians provided written consent.

## 3 RESULTS

Of the 79 scenarios tested, 61 increased the proportion of men and youth tested in the past year compared to the baseline PITC scenario (in which PITC is offered at approximately 50% of visits) (Table 3). The scenario that had the greatest increase in the proportion of men and youth tested was scenario 8, offering facility‐based HIVST all 12 months to both clients and guardians (and offering PITC to those who refuse HIVST, regardless of time since the last HIV test), resulting in as much as a 79% increase in those tested within the past 12 months, from 46% baseline to 83% testing coverage. The next most effective scenario (scenario 4) was PITC for clients and guardians at high coverage levels (>40% coverage) for 9 months of the year combined with three high OPD volume months of facility‐based HIVST for men and youth clients and their guardians—resulting in up to a 77% increase in proportion tested (46% baseline to 81% tested within 12 months). High levels of PITC coverage (>40%) for both clients and guardians year‐round (scenario 2) can result in a similar proportion of men and youth tested—as compared to a facility‐based HIVST scenario (scenario 8); however, PITC‐only scenarios with high coverage also require large increases in personnel cost to administer additional tests (up to a 174% increase).

**Table 3:** Proportion of men and youth tested and cost per new positive identified, by testing scenario, at different price points of the HIV self‐test kit

Scenario description					Percent change in the number of men and youth tested compared to baseline	Percent change in the number of healthcare workers required compared to baseline	Cost per positive identified at different HIVST kit prices		
Scenario	Percent of visits covered with PITC	Person targeted PITC	HIVST Number of months implemented	Person targeted HIVST			$1.80	$1.40	$1.00
1	5%	Client	–	–	–85.2%	–87%	$63		
	10%	Client	–	–	–72.2%	–78%	$66		
	15%	Client	–	–	–60.6%	–70%	$70		
	20%	Client	–	–	–49.0%	–57%	$72		
	30%	Client	–	–	–29.9%	–39%	$79		
	40%	Client	–	–	–13.8%	–17%	$85		
	*50%*	Client	–	–	0.0%	0%	$91		
	60%	Client	–	–	11.8%	22%	$98		
	70%	Client	–	–	22.0%	43%	$104		
	80%	Client	–	–	31.2%	61%	$111		
	90%	Client	–	–	39.2%	83%	$118		
2	5%	Both	–	–	–77.8%	–83%	$64		
	10%	Both	–	–	–58.5%	–65%	$68		
	15%	Both	–	–	–41.9%	–52%	$72		
	20%	Both	–	–	–26.3%	–35%	$77		
	30%	Both	–	–	–1.5%	–4%	$86		
	40%	Both	–	–	18.5%	26%	$95		
	50%	Both	–	–	34.7%	57%	$104		
	60%	Both	–	–	47.6%	87%	$113		
	70%	Both	–	–	58.1%	117%	$124		
	80%	Both	–	–	66.8%	143%	$134		
	90%	Both	–	–	73.7%	174%	$144		
3	10%/15%^a^	Both	–	–	–51.7%	–61%	$70		
	10%/20%^a^	Both	–	–	–44.7%	–57%	$71		
	10%/30%^a^	Both	–	–	–31.9%	–43%	$73		
	10%/40%^a^	Both	–	–	–19.6%	–30%	$75		
	10%/50%^a^	Both	–	–	–8.1%	–22%	$77		
	10%/60%^a^	Both	–	–	2.7%	–9%	$80		
	10%/70%^a^	Both	–	–	12.5%	4%	$83		
	10%/80%^a^	Both	–	–	21.1%	17%	$85		
	10%/90%^a^	Both	–	–	29.7%	26%	$88		
4	5%	Both	3	Both	33.0%	–57%	$93	$78	$62
	10%	Both	3	Both	37.3%	–48%	$97	$82	$67
	15%	Both	3	Both	40.9%	–39%	$100	$86	$71
	20%	Both	3	Both	44.5%	–30%	$104	$89	$75
	30%	Both	3	Both	51.0%	–13%	$110	$97	$83
	40%	Both	3	Both	56.8%	9%	$117	$104	$91
	50%	Both	3	Both	61.9%	26%	$124	$111	$98
	60%	Both	3	Both	66.4%	43%	$131	$118	$106
	70%	Both	3	Both	70.0%	61%	$138	$126	$114
	80%	Both	3	Both	73.8%	83%	$145	$133	$121
	90%	Both	3	Both	76.8%	100%	$152	$140	$128
5	5%	Client	3	Guardian	2.4%	–61%	$111	$93	$74
	10%	Client	3	Guardian	10.8%	–52%	$111	$94	$77
	15%	Client	3	Guardian	18.1%	–39%	$112	$96	$80
	20%	Client	3	Guardian	25.1%	–30%	$113	$98	$83
	30%	Client	3	Guardian	36.7%	–9%	$117	$103	$89
	40%	Client	3	Guardian	46.2%	9%	$121	$108	$95
	50%	Client	3	Guardian	54.3%	30%	$127	$114	$102
	60%	Client	3	Guardian	61.0%	48%	$132	$121	$109
	70%	Client	3	Guardian	66.8%	70%	$139	$127	$116
	80%	Client	3	Guardian	71.5%	87%	$145	$134	$123
	90%	Client	3	Guardian	76.1%	109%	$152	$141	$130
6	5%	Both/Client^b^	3	Guardian	–34.1%	–74%	$80	$69	$58
	10%	Both/Client^b^	3	Guardian	–20.8%	–61%	$82	$72	$63
	15%	Both/Client^b^	3	Guardian	–8.9%	–48%	$84	$76	$68
	20%	Both/Client^b^	3	Guardian	1.6%	–35%	$88	$80	$73
	30%	Both/Client^b^	3	Guardian	19.0%	–9%	$95	$89	$83
	40%	Both/Client^b^	3	Guardian	33.5%	22%	$103	$97	$92
	50%	Both/Client^b^	3	Guardian	45.0%	43%	$111	$106	$101
	60%	Both/Client^b^	3	Guardian	54.7%	74%	$120	$115	$111
	70%	Both/Client^b^	3	Guardian	62.5%	96%	$129	$124	$120
	80%	Both/Client^b^	3	Guardian	69.2%	126%	$138	$134	$129
	90%	Both/Client^b^	3	Guardian	75.0%	152%	$147	$143	$139
7	5%	Both/Guardian^b^	3	Client	1.6%	–65%	$85	$72	$59
	10%	Both/Guardian^b^	3	Client	9.4%	–52%	$89	$76	$64
	15%	Both/Guardian^b^	3	Client	16.5%	–43%	$92	$81	$69
	20%	Both/Guardian^b^	3	Client	23.0%	–30%	$96	$85	$74
	30%	Both/Guardian^b^	3	Client	34.9%	–9%	$103	$93	$83
	40%	Both/Guardian^b^	3	Client	44.5%	13%	$110	$101	$91
	50%	Both/Guardian^b^	3	Client	53.0%	35%	$118	$109	$100
	60%	Both/Guardian^b^	3	Client	60.0%	61%	$125	$117	$109
	70%	Both/Guardian^b^	3	Client	66.2%	83%	$133	$125	$117
	80%	Both/Guardian^b^	3	Client	71.2%	104%	$141	$133	$125
	90%	Both/Guardian^b^	3	Client	75.8%	126%	$149	$142	$134
8	0%	N/A	12	Both^c^	60.2%	–70%	$65	$54	$42
	0%	N/A	12	Both^d^	76.0%	–59%	$85	$69	$54
	0%	N/A	12	Both^e^	78.7%	–48%	$113	$92	$72
	0%	N/A	12	Both^f^	79.4%	26%	$483	$393	$302

Note: Current baseline scenario, as implemented.

^a^ The 10% coverage of visits is in the nine least busy months in the OPD, and the second number represents coverage in the most busy three months at the OPD.

^b^ During the self‐test months, the client would access PITC and the guardian would access HIVST (scenario 6), and the guardian would access PITC and the client would access HVIST (scenario 7).

^c^ Only can access a self‐test if have not reported an HIV test in the last 12 months.

^d^ Only can access a self‐test if have not reported an HIV test in the last 6 months.

^e^ Only can access a self‐test if have not reported an HIV test in the last 3 months.

^f^ Self‐test provided at all OPD visits regardless of the last time tested.

Among the HIVST‐only scenarios, only testing those not tested in the past 12 months (Ministry of Health guidelines) would reduce testing coverage to 74% of individuals being tested within a 12‐month window (vs. 83% with no restrictions). The reduction is largely due to when individuals’ visit health facilities and not returning after they would become eligible for testing (i.e. >12 months since the last test). Limiting testing to those who have not been tested in the past 6 months increased the proportion tested to 81% of individuals getting tested within a 12‐month window. There was limited marginal gain when further loosening testing eligibility criteria (82% and 83% of people tested when limiting testing to those who have not tested in the past 3 months and with no time restrictions on past testing, respectively).

**Figure 1:**
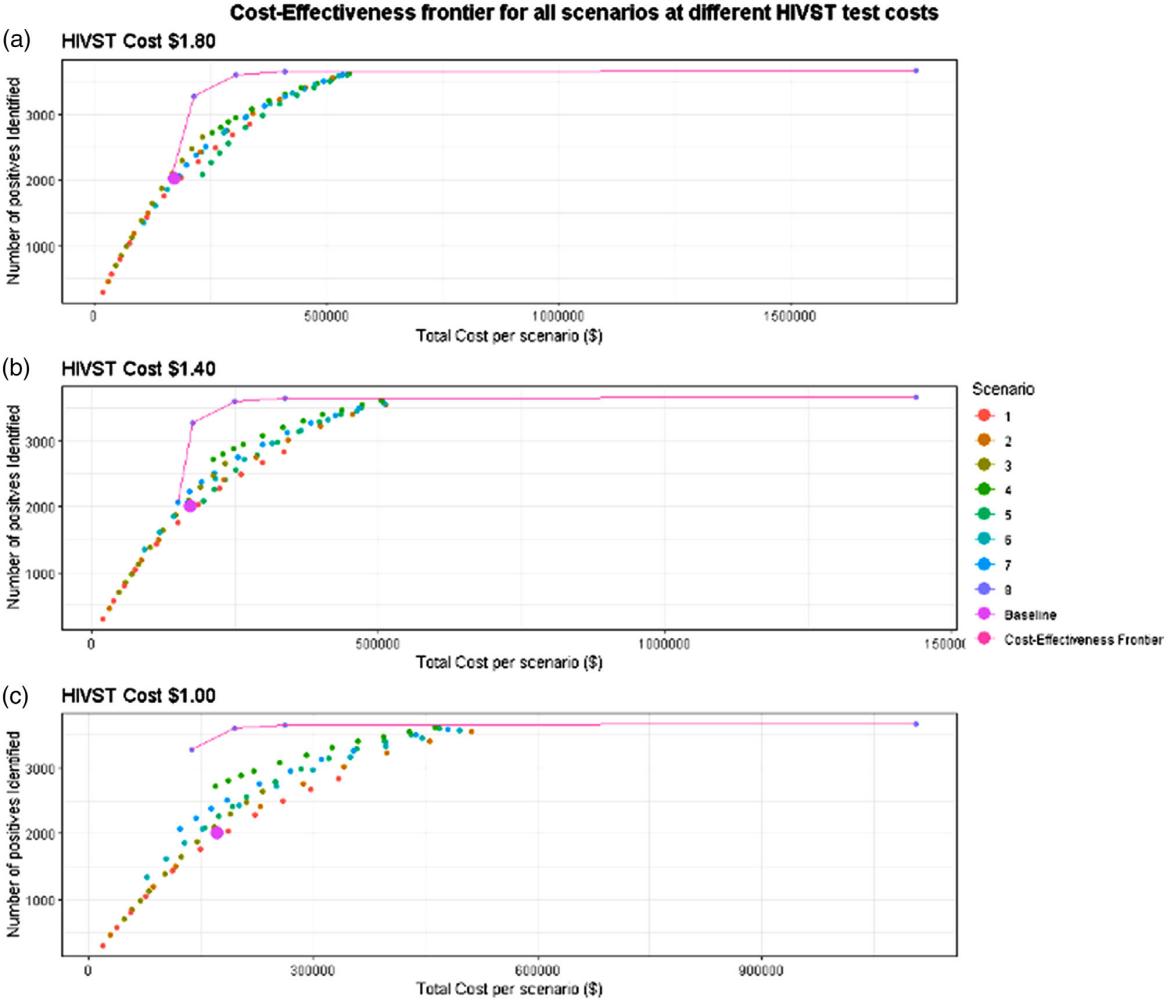
Cost‐effectiveness frontiers for all modelled scenarios at three different levels of HIVST test cost. All scenarios on the cost‐effectiveness frontier are further described in Table 4.

At the highest HIVST kit price ($1.80), the only cost‐saving scenario was where PITC covers 10% of all OPD client visits at random (for clients and guardians) and increases to 60% of all visits receiving PITC at random during the highest volume 3 months of the year (scenario 3, Table 4 and Figure 1). The subsequent four scenarios on the cost‐effectiveness frontier were all variations of scenario 8 (i.e. 12 months of HIVST offered at the health facility): year‐round HIVST offered, limited to those who have not tested in the last 12 months ($39/additional positive identified), limited to those who have not tested in the last 6 months ($262/additional positive identified), the last 3 months ($1983/additional positive identified) and not limited by time since the last test ($84,592/additional positive identified).

**Table 4:** Testing scenarios that are on the cost‐effectiveness frontier at different levels of HIVST kit price

Scenario	Percent of visits covered with PITC	Person targeted PITC	HIVST Number of months implemented	Person targeted HIVST	Total cost	Total positives identified^a^	Incremental cost‐effectiveness ratio
Price of HIVST kit= $1.80							
3	10%/60%	Both	–	–	$1,67,741	2095	Cost‐saving
1	*50%*	*Client*	–	–	$1,72,407	2011	Baseline^b^
8	0%	N/A	12	Both^c^	$2,13,407	3269	$39
8	0%	N/A	12	Both^d^	$3,03,550	3591	$262
8	0%	N/A	12	Both^e^	$4,11,443	3646	$1983
8	0%	N/A	12	Both^f^	$17,69,456	3662	$84,592
Price of HIVST kit= $1.40							
7	5%	Both/Guardian	3	Client	$1,49,131	2073	Cost‐saving
1	*50%*	*Client*	–	–	$1,72,407	2011	Baseline^b^
8	0%	N/A	12	Both^c^	$1,75,794	3269	$22
8	0%	N/A	12	Both^d^	$2,49,355	3591	$228
8	0%	N/A	12	Both^e^	$3,37,161	3646	$1614
8	0%	N/A	12	Both^f^	$14,38,165	3662	$68,583
Price of HIVST kit= $1.00							
8	0%	N/A	12	Both^c^	$1,38,181	3269	Cost‐saving
1	*50%*	*Client*	–	–	$1,72,407	2011	Baseline^b^
8	0%	N/A	12	Both^d^	$1,95,160	3591	$177
8	0%	N/A	12	Both^e^	$2,62,878	3646	$1245
8	0%	N/A	12	Both^f^	$11,06,874	3662	$52,573

^a^ Assuming 2.5% positivity.

^b^ Not on the cost‐effectiveness frontier.

^c^ Only can access a self‐test if have not reported an HIV test in the last 12 months.

^d^ Only can access a self‐test if have not reported an HIV test in the last 6 months.

^e^ Only can access a self‐test if have not reported an HIV test in the last 3 months.

^f^ Self‐test provided at all OPD visits regardless of the last time tested.

At the second highest HIVST kit price ($1.40), the scenario in which PITC covers 5% of all visits at random during the year for both clients and guardians and provides HIVST to clients only during the highest volume 3 months of the year for clients only (not guardians) (scenario 7) is considered cost‐saving compared to baseline PITC. Similarly to when the HIVST kit price was $1.80, the subsequent four scenarios on the cost‐effectiveness frontier were all sub‐scenarios of scenario 8 (i.e. 12 months of HIVST offered at the health facility): limited to those not tested in the last 12 months ($22/additional positive identified), the last 6 months ($228/additional positive identified), the last 3 months ($1614/additional positive identified) and not limited by time since the last test ($68,583/additional positive identified).

At the lowest HIVST kit price ($1.00), the scenario in which HIVST is offered for 12 months (limited to those not tested for HIV in the previous 12 months, scenario 8) was considered cost‐saving compared to baseline PITC, resulting in a 20% reduction in total costs compared to the current coverage of PITC alone. The subsequent three scenarios on the cost‐effectiveness frontier were the remaining versions of scenario 8: limited to those not tested in the last 6 months ($177/additional positive identified), the last 3 months ($1245/additional positive identified) and not limited ($52,573/additional positive identified).

## 4 DISCUSSION

In this data‐driven, individual‐based model, we assessed the cost‐effectiveness of multiple implementation strategies at different HIVST price points for HIV testing in OPD settings in Malawi that would increase testing coverage among men and youth. We found that almost all facility‐based HIVST scenarios increase community‐level HIV testing coverage among men and youth when implemented in OPD settings. Men and youth are historically underserved by current PITC programmes, as standard PITC achieves poor coverage within busy OPD settings [6, 17]. The overall increase in community‐level testing coverage is possible due to the high frequency in which men and youth visit health facilities either as clients or guardians, but have not typically been offered testing services [18]. Implementing year‐round HIVST in OPD settings was highly cost‐effective in most scenarios‐ and cost‐saving compared to the baseline PITC (as much as a 19.7% reduction in cost compared to baseline) when the price of the HIVST kit is reduced to $1.00. Modelled costs per positive client identified in these scenarios (at the $1.00 test cost) ranged between $42 and $302 for all year‐round HIVST scenarios which encompasses some of the mean costs seen in other facility‐based HIVST studies ($80/positive identified [19], $97.50/positive identified [20] and $100–$200/positive identified). Further, it is worth noting that in scenarios which limit testing (based on the last test), the modelled costs range between $42 and $72, which is notably lower than in other studies.

The only PITC‐based scenarios that can increase the proportion of men and youth tested to the same degree as year‐round HIVST require a significant increase in personnel requirements. The reduction in healthcare worker time required for HIVST compared to the current status quo is a significant advantage to the HIVST scenarios, requiring up to 70% fewer personnel than the current standard of care. Saved human resource capacity from HIVST implementation could be used for other activities like patient linkage, treatment support, to provide support elsewhere in the facility or to additional testing strategies, such as index testing or initial HIV screening to increase testing yield.

Our findings contribute to a growing body of evidence on how facility‐based HIVST can contribute to achieving the UNAIDS first 95 goals for those traditionally unreached by HIV services. HIVST is consistently shown to increase testing rates in comparison with standard PITC models, even in randomized controlled trials where providers receive additional support for implementing PITC [11, 12, 14, 21]. There is consensus that HIVST is associated with a higher absolute number of patients on treatment for HIV, although linkage rates differ based on the HIVST implementation [11, 14, 21] and antiretroviral treament (ART) initiation has been lower among those diagnosed through self‐testing in comparison to PITC [14, 22]. Hybrid facility‐based PITC + HIVST strategies may allow health facilities to maximize the benefits of both modalities, increasing coverage and reducing personnel costs without sacrificing quality of and linkage to care. It is important to consider that for many of the hybrid strategies to be cost‐effective, there is often a necessity to reduce PITC coverage. This may be an implementation obstacle if it appears as though there is backtracking on progress for the provision of HIV testing services. Further, if PITC were scaled back and HIVST was poorly implemented, the overall test coverage could drop. Feasible implementation of facility‐based HIVST would thus likely need a phased approach where it is initially implemented alongside standard of care before being merged into the cost‐effective scenarios shown in our analysis.

Our study has several limitations. First, for different standard PITC coverage levels, we randomly assigned individuals to be offered an HIV test, and the uptake of that test was dependent on their survey response. In reality, PITC coverage among outpatient visits is unlikely to be completely random. Any selection in favour of someone at increased risk of HIV infection would result in increased cost‐effectiveness of any scenarios, including PITC, through a reduced cost per positive test. However, the sensitivity of any type of screening needs to be weighed against the additional time required to administer it, which could increase human resource requirements and offset the reduction in cost per positive test. Within our test assignment, we also did not offer HIVST to PITC refusers given our assumptions about implementation from the facility‐based HIVST trial [16]. Future work may need to consider the impact of initial PITC refusal and implementation strategies for offering HIVST to those who decline PITC. Second, the model was based on self‐reported survey data. Participants in the survey may have under or overestimated the number of visits to the health facility. This may impact the total estimated proportion of men and youth who would receive an HIV test, but not differentially by scenario. Third, we have assumed a uniform distribution of positive cases across all scenarios to calculate a cost per positive identified, to ensure comparability to the literature. While it is possible that some scenarios are more/less likely to identify new positives with fewer tests, given that the target population is in every instance identical (men and youth in the OPD), the yield is unlikely to differ meaningfully. Finally, due to people reporting health facility visits retrospectively, it is likely that dates of more recent visits are more precise as compared to visits that were further in the past. This was circumvented by truncating results to the past 12 months. Additionally, instead of modelling specific months, we modelled low‐ and high‐volume OPD months to simulate coverage in either type of scenario.

## 5 CONCLUSIONS

Facility‐based HIVST in the OPD is cost‐effective and can significantly increase access to HIV testing for men and youth in Malawi. The feasibility of covering all OPD visits with HIVST will depend on the available budget for test kits. Additional investment in capacity to implement year‐round facility‐based HIVST, limiting testing based on time‐since‐last‐HIV‐test and introduction of lower‐priced HIVST products should be prioritized to maximize the impact of facility‐based testing strategies.

## COMPETING INTERESTS

The authors declare that there are no competing interests.

## AUTHORS’ CONTRIBUTIONS

BEN, ND and KD conceived the study. KB, MM, SK, CS, ND and KD acquired and analysed data for the model. BEN, AdN, MB and GR developed the model. BEN, AdN, CWC, CAR and KD interpreted model results. BEN, AdN and KD wrote the first draft of the manuscript. MB, KB, MM, GR, CWC, SK, CS, CAR and ND contributed to the writing of the manuscript. All authors read and approved the final manuscript.

## FUNDING

The Foreign, Commonwealth and Development Office of the United Kingdom of Great Britain and Northern Ireland funded the study (grant number: 300380), as well as the United States Agency for International Development (Cooperative agreement 72061221CA00010). The funders had no role in study design, data collection and analysis, decision to publish or preparation of the manuscript.

## Data Availability

Data sharing is not applicable—no new data are generated, or the article describes entirely theoretical research.
